# Observations on the Treatment of Scarlatina Anginosa at the Asylum for Female Orphans, Westminster, and at the Friend's Boarding School at Ackworth, Yorkshire

**Published:** 1814-12

**Authors:** Samuel Fothergill

**Affiliations:** Craven-street


*For the Medical and Physical Journal.*

### *Observations on the Treatment of Scarlatina Anginosa at the Asylum for Female Orphans, Westminster, and at the Friend's Boarding School at Ackworth, Yorkshire;* by SAMUEL FOTHERGILL, M.D.

THE history of Scarlatina Anginosa has been so often and so well detailed, that little remains for future writers to add. It is also generally admitted that the varieties of the disease are derived from one common origin; that persons exposed to the same source and form of contagion shall have the disease variously; in some it may appear as scarlatina, without any affection of the throat; in others the tonsils shall be inflamed, or even ulcerated; and in some the most malignant symptoms may occur. These facts are best elucidated by the appearance of the disease in large schools, or other institutions, where all the varieties of the disorder can readily be traced.

In some cases it is not difficult to assign, as a probable cause for any peculiar severity or malignancy of the complaint, a particularly gross habit in the patient attacked, a constitution affected by previous illness, irregular and improper diet, and other causes which the physician deems adequate to account for a contagious disease like scarlatina proving unusually severe. But in many instances the fever occurs in different forms without any thing sufficiently prominent in the habit of the patient to account for the degree of intensity, whether small or great, which it assumes. Sydenham, indeed, seems to accuse the physician of sometimes being accessory, by the activity of his practice, to the fatality of the disease. Those practitioners who still pursue what is called the cordial plan of treatment, either are ignorant of what that great physician wrote, or they must call in question, what is least to be doubted, his fidelity and accuracy. His remarks are not inapplicable in the present age: "Si plus negotii ægris facessamus, vel lectulis continenter incarcerando, vel cardiacis aliisque supervacaneis nimis docte, et (ut vulgo videtur) secundum artem supra modum ingestis, morbus statim intenditur, et æger non raro nulla alia de causa, quam *nimia medici diligentia*, ad plures migrat."*

Where the disease is disposed to proceed favourably, this injudicious interference of the physician is truly lamentable. It is equally lamentable when he becomes alarmed the instant that the throat appears ulcerated, and the tongue furred; and still more so if he imagines that these symptoms indicate the necessity of resorting to bark, wine, and other warm stimulating medicaments. When such symptoms have occurred early in the complaint, I have rarely seen any benefit arise from the use of bark or wine, either separate or combined. We are too apt, if a patient struggles through a dangerous disorder, to attribute his recovery to our medicines. Some years ago, I gave wine and bark in severe cases of scarlatina anginosa, but I seldom, upon the most attentive

* Sydenham Opera, 8vo. p. 261.

watching, watching, could perceive any good effect result from such treatment, and have since altogether abandoned it, until the fever is removed, when occasionally it may be serviceable.

In the months of September and October last, about fifty-five children, at the Asylum for Female Orphans, of various ages, from ten years to fifteen, with two adults, were affected with scarlet fever and sore-throat. In some of them the throat was very slightly affected; in others it was ulcerated early in the complaint. Although most of the cases were slight, in a few of the children, especially those whose throats were ulcerated, the fever was considerable. The tongue coated with a thick dark-coloured fur, and a great prostration of strength indicated the serious, if not malignant, character of the disease.

The treatment pursued was extremely simple: the instant a child was observed to droop, and appear sickly, she was removed from the school to a part of the establishment appropriated as an infirmary, where great care was observed to ventilate the chambers. A purgative was administered and repeated as circumstances indicated. In most of the cases it consisted of calomel and rhubarb, in doses adapted to the age of the patients; in slight cases, sulphate of magnesia was given; and, in those of a severe form, to the calomel and rhubarb were added small doses of antimonial powder. Infusion of roses acidulated, was ordered when the opening medicines were not operating. The whole surface of the body, when hot, was well sponged with cold water. In some of the cases acid gargles were used. Under this treatment, which was strictly enjoined by the attending apothecary, Mr. Heaume, and faithfully observed by the nurse, I observed, on my occasional visits, the children proceed uniformly well. Neither wine, bark, or cordials of any description, were given till after the fever had entirely ceased; and the whole of the patients recovered without any unpleasant secondary consequences so frequently attendant on the disease.

Upon looking into Dr. Willan's great work on Cutaneous Diseases, in which scarlatina occupies a large space, I was rather surprised at the following paragraph on the effect of purgatives in this disease. "Purgatives (he remarks) have nearly the same debilitating effect as blood-letting. They are, indeed, very seldom necessary, for though a few patients may, on the first day, be affected with bilious vomiting and diarrhœa, the state of the bowels is more uniform than in other febrile complaints. It has also been remarked that no hardness or tension of the abdomen occurs at an early period of the disease." I certainly have never witnessed the debilitating effect from purgatives, and should much doubt Dr. Willan himself having experienced it, especially when in a note to the passage just quoted, he informs us that a small dose, as two or three grains, of calomel is very useful; to which, on the first attack of the disease, he usually added an equal portion of antimonial powder.

Dr. Willan has displayed much erudition upon the subject of scarlet fever, but the practice recommended by the different authors whom he quotes, is so various and opposite, that perhaps he would have conferred more benefit on the profession if he had confined himself to the result of his own observation, or at least have pronounced decidedly upon the merits of the several modes of treatment recommended. Amid the doubts of cautious, and the errors of bold practitioners, surrounded by conflicting opinions whenever he consults books, till he has attained experience of his own, it is very difficult for the young practitioner to decide; but he may on most occasions, whatever practice he pursues, find authority to shelter him. Thus, in scarlatina anginosa, without taking the trouble to consult any other works than that of Dr. Willan, we may find authority for bleeding and for not bleeding; for giving emetics and purgatives, or for omitting them; for giving acids or alkalies, saline medicines, or tonics; for using cold or warm affusion; in short, every remedy is advocated.

Dr. Willan seems to quote with approbation the treatment pursued by Dr. Binns, at Ackworth School, where, in the space of four months, 171 individuals were affected with the complaint, of which the following table gives the result:—

| *Affected with the Disease.* | | *Died.* |
|---|---|---|
| Boys | 105 | 5 |
| Girls | 49 | 2 |
| Men | 8 | 0 |
| Women | 9 | 0 |
| | 171 | 7—nearly 1 in 24. |

Dr. Binns plan was to give an emetic in the beginning, and small doses of calomel; but he is anxious to inform us that "particular care was taken to support the patient during the operation." The nature of that support may be readily imagined upon reading the following extract. "As soon as the stomach was settled after an emetic, I generally began with the following mixture:

℞. Pulv. Cinchon. Unc. j.
Pulv. Aromat. dr. ij.
Vini Rubri, ℔ij.

When this had been used about *six weeks*, I substituted for the the aromatic powder some canella alba, with one drachm of powdered ginger, which seemed to answer equally well. A child of ten years old would take about three table-spoonfuls of the mixture four, five, or six times, in twenty-four hours. Those who were very ill, took in addition from a pint to a pint and a half, or even more, of red port wine, or of a composition,* which was not unpalatable, which agreed well with the children, and was a considerable saving to the institution. Another remedy was an infusion of red rose-buds in decoction of Peruvian bark, sweetened with extract of liquorice, and acidulated with vitriolic acid: to this was occasionally added, simple or compound tincture of bark. In extreme cases, the extract and compound tincture of bark, with a little ammonia, and sometimes aromatic confection, were made into a mixture with peppermint water, a dose being given every half hour. Such as were but slightly affected, generally drank treacle-beer made with ginger in it, small ale, and sometimes cyder, sage tea acidulated with vitriolic acid, and almost every other drink that was agreeable to them. New milk, and every nutritious food, both animal and vegetable, were allowed as the patients could take them. It is impossible to specify with certainty the quantity of wine taken by individual patients, but, from the general consumption, when a number of bad cases occurred together, it appeared *that children about twelve years of age must have taken each a bottle of red port, and a bottle of raisin wine, in twenty-four hours, for several successive days.* Sometimes strong brandy and water, sometimes brandy unmixed, was given with comfort and advantage to the patient."

Such was the practice pursued at a large boarding school, where every attention was given to the children on the first attack of the disease. Notwithstanding its boasted efficacy, and that the proportion of deaths was only one in twenty-four, considering the tender age of the children, I see no sufficient inducement to give two bottles of wine, besides bark, aromatics, and other stimulants, in the course of twenty-four hours, for several successive days. On the contrary, I consider it a fortunate occurrence that the mortality amongst the children was not more considerable.

*Craven-street;* SAMUEL FOTHERGILL.

*Nov.* 13*th*, 1814.

---

* This was, red port 2 pints, raisin wine 6 pints, decoction of logwood 1 pint, alum 1 ounce, British brandy 8 table-spoonfuls.

